# Inside the black box of comparative national healthcare performance in 35 OECD countries: Issues of culture, systems performance and sustainability

**DOI:** 10.1371/journal.pone.0239776

**Published:** 2020-09-28

**Authors:** Jeffrey Braithwaite, Yvonne Tran, Louise A. Ellis, Johanna Westbrook

**Affiliations:** 1 Centre for Healthcare Resilience and Implementation Science, Australian Institute of Health Innovation, Macquarie University, Sydney, Australia; 2 Centre for Health Systems and Safety Research, Australian Institute of Health Innovation, Macquarie University, Sydney, Australia; Technical University of Kosice, SLOVAKIA

## Abstract

**Background:**

Is national healthcare performance associated with country-level characteristics, and if so what are the implications for international health policy?

**Methods and findings:**

We compared Hofstede’s six cultural dimensions against relative health systems performance of 35 countries. Hierarchical cluster analysis identified best-matched groupings of countries. Performance was measured by the Organisation for Economic Co-operation and Development’s (OECD’s) *Health at a Glance* indicators data framework (five dimensions with 57 indicators) and the United Nations’ (UNs’) Sustainability Development Goals (SDG) data set (15 indicators). Three country clusters emerged: Collective-Pyramidal (n = 9: comprising Slovak Republic, Mexico, Poland, Greece, Spain, Turkey, Portugal, Chile, and Slovenia); Collaborative-Networked (n = 12: UK, Canada, Australia, USA, Ireland, New Zealand, Netherlands, Finland, Iceland, Norway, Denmark, and Sweden); and Orderly-Future Orientated (n = 14: Korea, Estonia, Latvia, Austria, Israel, Japan, Czech Republic, Hungary, Italy, Belgium, France, Germany, Luxembourg and Switzerland). The Collaborative-Networked cluster had significantly better performing health systems measured by both the *Health at a Glance* and SDG performance data, followed by the Orderly-Future Orientated cluster, followed by the Collective-Pyramidal cluster. The Collaborative-Networked Cluster was characterized by low power distance (e.g., greater levels of equity), low uncertainty avoidance (e.g., toleration of others’ opinions), individualism (e.g., self-reliance) and indulgence (e.g., drives and norms to enjoy life and have fun).

**Conclusions:**

National cultures are associated with healthcare performance on two key international measures. In national and international efforts to improve health system performance, cultural characteristics play an important role. This information may be of value to regulators, policymakers, researchers and clinicians examining the practical impact of culture on healthcare performance.

## Introduction

It is a considerable challenge to assess the performance of entire health systems and judge their relative contributions to the health of populations. Health systems are complex, multi-faceted entities with many internal and external variables. Recent work has attempted to benchmark countries by ranking them according to aggregated measures of national health system performance. The latest advances include those of the Commonwealth Fund, ranking up to 19 countries across measures such as quality of care, spending, utilisation and governance [1], the World Health Organisation’s (WHO’s) data on all countries via its [2] and the Organisation for Economic Co-operation and Development’s (OECD’s) *Health at a Glance* indicators data framework [3, 4]. Other work has taken a country-level case study approach to identify national health system strengths, and then drawn together the findings from 30, 60 and then 152 countries into a strategic overview of performance [5].

There are notable challenges to assessing relative health system performance. These include establishing criteria for selecting the mix of indicators to incorporate into the overall measure; the apples and oranges problem of achieving standardisation so that benchmarking is calibrated to the same comparative variables; and the attribution problem, determining which indicators or variables are most or least influential in determining performance.

One under-researched aspect of national performance is the extent to which healthcare performance is a reflection of national culture [6]. What association holds between the two constructs? Does US individualism and Japanese collectivism, to provide just two examples, influence relative national health systems performance—and if so, in what ways? We define natural culture as the distinctive aggregated patterning of collective behaviours, practices, rituals, routines, values, attitudes, ideologies and propensities that characterise and differentiate one nation from another [7–10] and health systems performance as the teleologically-oriented activities, inputs, processes, outputs and outcomes that are deployed to meet implicit and explicit healthcare, patient, clinical, organisational, and systems-wide productivity goals [4, 11, 12].

Despite us having synthesised much prior work into these overarching definitions, we note that both national culture and health systems performance are multi-dimensional constructs. Work to date to measure their dimensionality is now sufficiently mature to warrant in-depth study of their relationship. In essence, we now have a better understanding about their aggregate characteristics and the components that underly them. We turn to a discussion of the elements that undergird each construct.

### National culture

National culture is a multi-layered phenomenon [7–10, 13]. Many studies have used Hofstede’s cultural dimensions, originally developed from research in one large multinational business corporation (IBM Corp) in 40 countries [14] and later extended to 76 countries [15]. In the employee surveys of these countries, Hofstede found distinct patterns of cultural expression and practices. He homed in simply on national culture as “the collective programming of the mind which distinguishes the members of one group or category of people from others” [15]. From this perspective, and our definition above, cultural dimensions are believed to represent, at a macro level, the patterned thinking and shared behaviours between large social groups, and on a micro level, the behaviour of small collectives [16]. Important variations between countries on Hofstede’s dimensions have been documented [14, 15, 17].

Noort et al. [18] proposed that national cultures can influence outcomes in localised settings such as hospitals. For example, national cultural values were found to be significantly related to hospital safety culture. Both antibiotic prescribing behaviour and infection control behaviour have also been associated with cultural characteristics [19–22]. Deschepper and colleagues (2008) found the cultural dimension ‘power distance’ to be squarely associated with antibiotic use, showing that the different ways that patients in 27 European nations deal with authority is a factor in how antibiotics are prescribed and taken in those countries.

Mackenbach and McKee [17] examined associations between cultural values and population health in 42 European countries, and found differences in cultural values accounted for striking variations in health behaviour even between neighbouring countries in Europe. That study suggested that health behaviours and health outcomes are partly determined by–or strongly related to–variations in culture [17].

### Healthcare performance

Healthcare performance is also multi-dimensional. The OECD’s *Health at a Glance 2017*: *OECD Indicators* report [4] provides a compendium of aggregated data drawn from a basket of indicators on the health status of country-level populations and health systems performance. In the OECD’s framework, performance is a product of, and should be assessed against, factors such as health status; risk factors for health; access to care; quality of care; health expenditure and financing; healthcare workforce; and healthcare activities, within the demographic, economic and social context of each country. In parallel work the UN’s Sustainable Development Goals (SDGs), comprising 17 universal goals, 169 targets and 230 indicators, measured progress in 188 countries against 33 of those indicators, with the aim of understanding “gains” and “gaps” to improve the health of populations and to rank comparative country performance. Translation of the SDG framework into investments and policy, on the other hand, remains in its infancy [23]. It is also important to acknowledge that the performance of healthcare systems is mediated by institutions (Health Ministries, Government policy, population groupings for administrative purposes, hospitals and hospital chains), and the like [2, 3, 24].

### Aims and purpose of the study

The purpose of this research is to describe the relationships between cultural influences on healthcare systems performance.

## Methods

### Countries

We included all OECD member countries at December 2017 (n = 35): Australia, Austria, Belgium, Canada, Chile, Czech Republic, Denmark, Estonia, Finland, France, Germany, Greece, Hungary, Iceland, Ireland, Israel, Italy, Japan, Korea, Latvia, Luxembourg, Mexico, Netherlands, New Zealand, Norway, Poland, Portugal, Slovak Republic, Slovenia, Spain, Sweden, Switzerland, Turkey, United Kingdom (UK), and United States of America (USA).

### Measuring national culture

Hofstede’s cultural dimensions were developed initially based on factor analysis of attitude surveys among employees of IBM, and he later applied the characteristics he found to the nations from which the employees came. The six cultural dimensions [15, 25–27] are summarised in Box 1.

**Box 1. Hofstede’s six cultural dimensions**

**Power distance (PD) index:** defined as the propensity for the less powerful to accept that power is distributed unequally, essentially measuring the extent of hierarchies in a society. A high index score indicates acceptance of inequality in power, whereas a low index score signifies people questioning authority and attempts to re-distribute power.

**Individualism vs Collectivism (IV):** the degree members of a culture are integrated into groups. Individualism manifests in societies in which the ties between people are loose, that is, everyone is expected to look after themselves or their immediate family members. Collectivism is more prevalent in societies in which people from birth onwards are assimilated, belonging to cohesive groups which tend across the lifespan to protect members in exchange for in-group loyalty.

**Masculinity vs Femininity (MF):** The masculine-oriented societies are considered assertive and competitive whereas the feminine-oriented societies are considered modest and caring. Women in societies that are feminine have similar modest and caring values as men, whereas in masculine societies women are somewhat assertive and competitive but in aggregation, not to the same extent as men.

**Uncertainty avoidance (UA) index:** the extent that societies tolerate ambiguity. Members of uncertainty-avoiding cultures feel threatened by ambiguous unstructured situations and try to minimise this by opting for strict behavioural codes. Uncertainty-accepting cultures are more tolerant of opinions that are different to their own and what is different is considered curious; and they also have fewer rules.

**Long-term orientation vs Short-term orientation (LT):** how societies maintain links with their own past while handling the challenges of the present and future. A long-term orientation is represented in societies that foster and orient towards future rewards, particularly in terms of perseverance and thrift. A short-term orientation prevails in societies that project virtues related to the past and present.

**Indulgence vs. Restraint (ID):** the extent to which people try to control their desires and impulses, based on the way they were socialised. Indulgence is represented in societies that allow relatively free gratification of basic and natural human drives related to enjoying life and having fun. Restraint manifests in societies that suppress gratification of desires and regulate it by means of often strictly imposed social norms.

Sources: derived from Hofstede [15, 25–27].

The national culture data for this study were collected from a publicly available source; the Hofstede’s cultural dimension data matrix (https://www.hofstede-insights.com/product/compare-countries/). All six cultural dimension scores were available for the 35 OECD member countries, except for the “indulgence vs restraint” score for Israel.

### Measuring health systems performance based on health dimensions

Healthcare system performance and outcomes data were obtained from two sources: the OECD’s healthcare indicators, and the United Nation’s (UN’s) Sustainable Development Goals’ data sets. First, we examined data in the OECD’s Health Care Quality Indicator Project, using the *Health at a Glance 2017* framework [3, 4]. *Health at a Glance 2017* presents a set of dashboards which are designed to shed light on how well OECD countries do in promoting the healthiness of their populations and improving their health system performance. The dashboards summarise some of the relative strengths and weaknesses of countries on a selected set of performance indicators to help identify priority areas for actions. Essentially, the dashboards highlight how well OECD countries are doing along five health dimensions, across 57 indicators: 1) health status (HS, 9 indicators, e.g., life expectancy, mortality); 2) risk factors to health (RF, 7 indicators, e.g., smoking, healthy lifestyle); 3) access to care (AC, 16 indicators, e.g., waiting times for elective surgery, unmet needs); 4) quality of care (QC, 20 indicators, e.g., avoidable hospital admissions, prescribing in primary care); and 5) healthcare resources (HCR, 5 indicators, e.g., health expenditure, out of pocket spending) [4].

We calculated standard z-scores for each of the health dimensions with individual standard scores calculated for each indicator. As each health dimension comprised at least five indicators, the average standard score was used as the overall score for that particular health dimension following the methodology of the Commonwealth Fund’s scorecards on health system performance [28]. Each of the five health dimensions was grouped into two categories for analysis: those performing at or better than the mean (0 or above in the average standard scores) and those performing below the mean (below 0 in the average standard score).

### Measuring health goals based on Sustainable Development Goals (SDGs)

Health outcomes were measured using SGD3, our second performance data set. This data source aims to “ensure healthy lives and promote well-being for all at all ages”. We accessed the 2017 SDG Index containing 33 health related indicators with 15 used to calculate the SDG3 index [23, 29]. SDG3 focusses on 13 health outcome targets with major focus on reproductive and child health, infectious and non-communicable diseases and health systems and funding. Some indicators used for the SDG3 index are similar to the indicators utilised for healthcare systems performance. However, the SDG index scores are presented in percentage form and can be interpreted as the percentage achievement in these health targets, providing an overall view of health outcome performance. The SDG dashboards provide indicators coloured as “green”, “yellow”, “orange” and “red”, indicating whether the country has already achieved the SDG goal, with “green” being achieved and “red” being far from achieved [29]. A green rating was only assigned if all indicators under a particular goal were met. We applied the SDG3 index and dashboard ratings as an overall indicator of countries’ ability to meet sustainable health outcomes.

### Statistical analysis

All statistical analyses were performed using SPSS version 25 and R version 3.5.2 using RStudio v1.1.442. The R packages NbClust R, dendextend and the hclust function were applied for hierarchical cluster analysis. Data-driven cluster analysis was conducted for the 35 OECD member countries based on their similarities or dissimilarities on the six cultural dimensions. To determine the relevant number of clusters for the six dimensions, clustering validity indices were tested for information such as intraclass compactness, interclass isolation, the geometric or statistical properties of the data, and similarity or dissimilarity properties, using the NbClust R package. Once the best number of clusters was determined, hierarchical clustering using the agglomeration method of complete-linkage was performed to group the countries that were most closely linked based on their six cultural dimension qualities. Other statistical analysis included descriptive analysis for the cluster groups, one-way ANOVA to determine differences for continuous scores and Fischer’s Exact test for differences in categorical groups. Fischer’s exact test was chosen for its validity for small sample sizes.

## Results

### Cluster analysis

**Fig 1:**
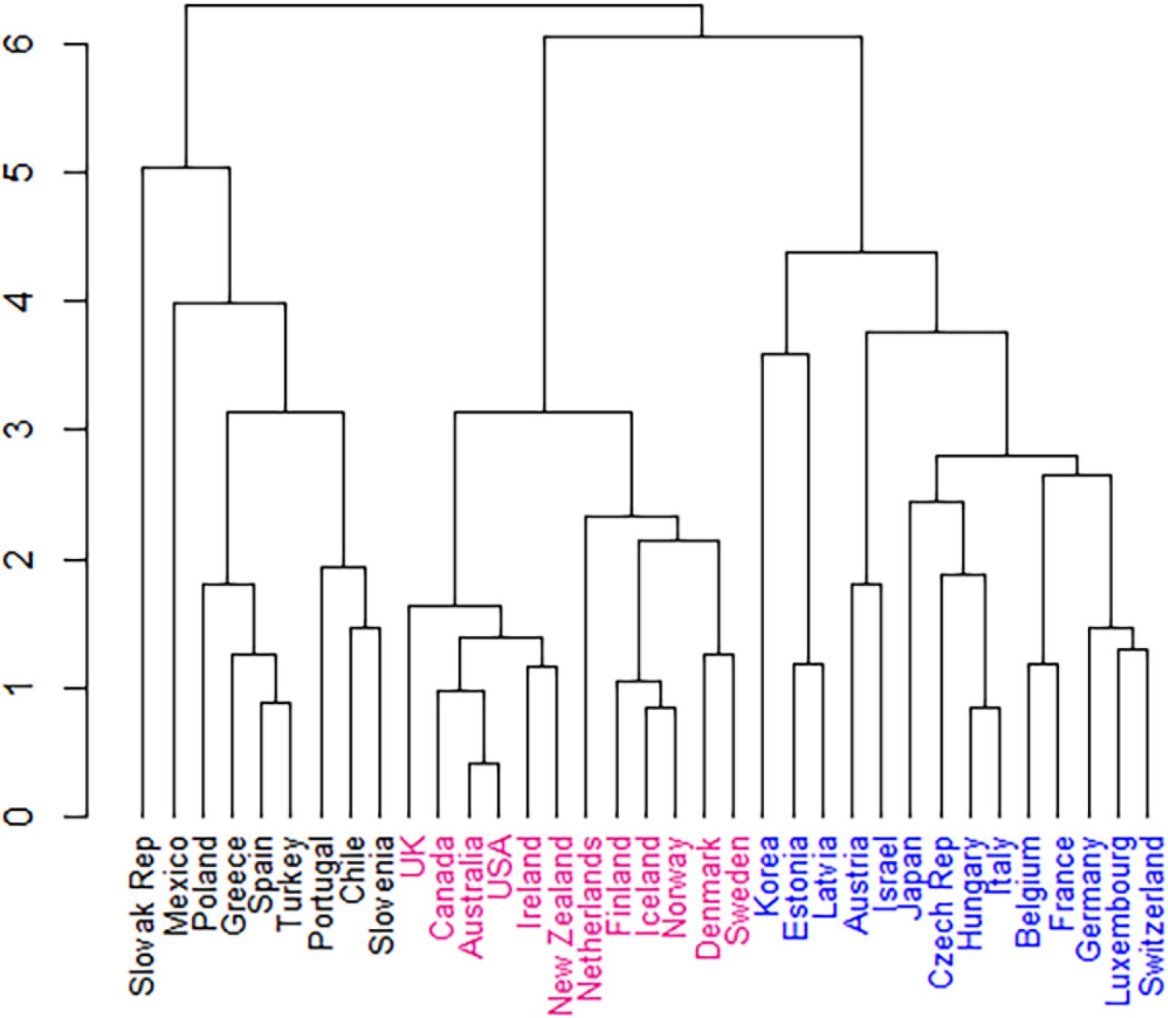
Dendrogram created from the hierarchical cluster analysis of countries by cultural dimensions.

From 23 indices tested, 13 clustering validity indices (the majority) proposed three clusters to be the optimal number of groups for the 35 OECD member countries, derived from their 6 cultural dimension features. Fig 1 shows the dendrogram results of the hierarchical cluster analysis.

**Fig 2:**
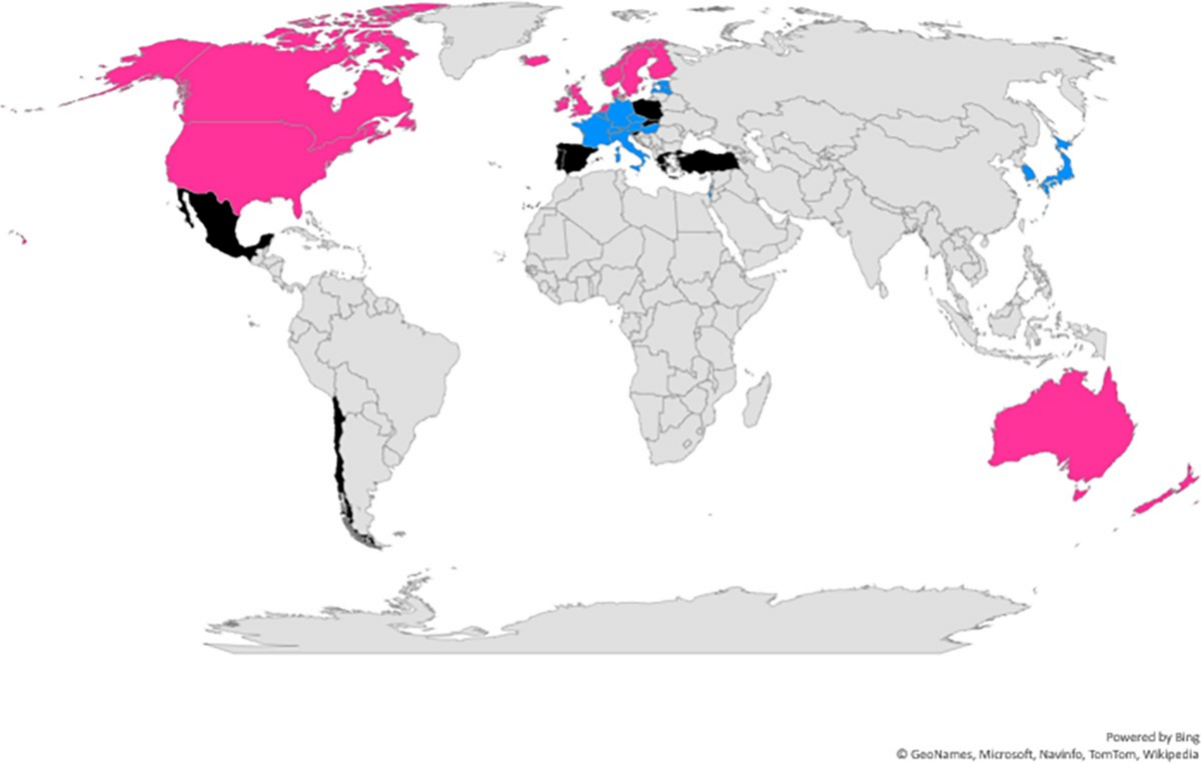
Geographical representation of the three cluster groups (The world map was generated in Microsoft 365 ® Excel ®).

The three clusters were identified when the branches were cut at height six. Cluster 1 (black) comprised nine countries: the Slovak Republic, Mexico, Poland, Greece, Spain, Turkey, Portugal, Chile, and Slovenia. Cluster 2 (pink) comprised 12 countries: UK, Canada, Australia, USA, Ireland, New Zealand, Netherlands, Finland, Iceland, Norway, Denmark, and Sweden. Cluster 3 (blue) comprised 14 countries: Korea, Estonia, Latvia, Austria, Israel, Japan, Czech Republic, Hungary, Italy, Belgium, France, Germany, Luxembourg and Switzerland. Fig 2 shows the geographical representation of the cluster groups. This illustrates that clustering based on cultural dimensional qualities does not strongly reflect geographical proximity.

**Fig 3:**
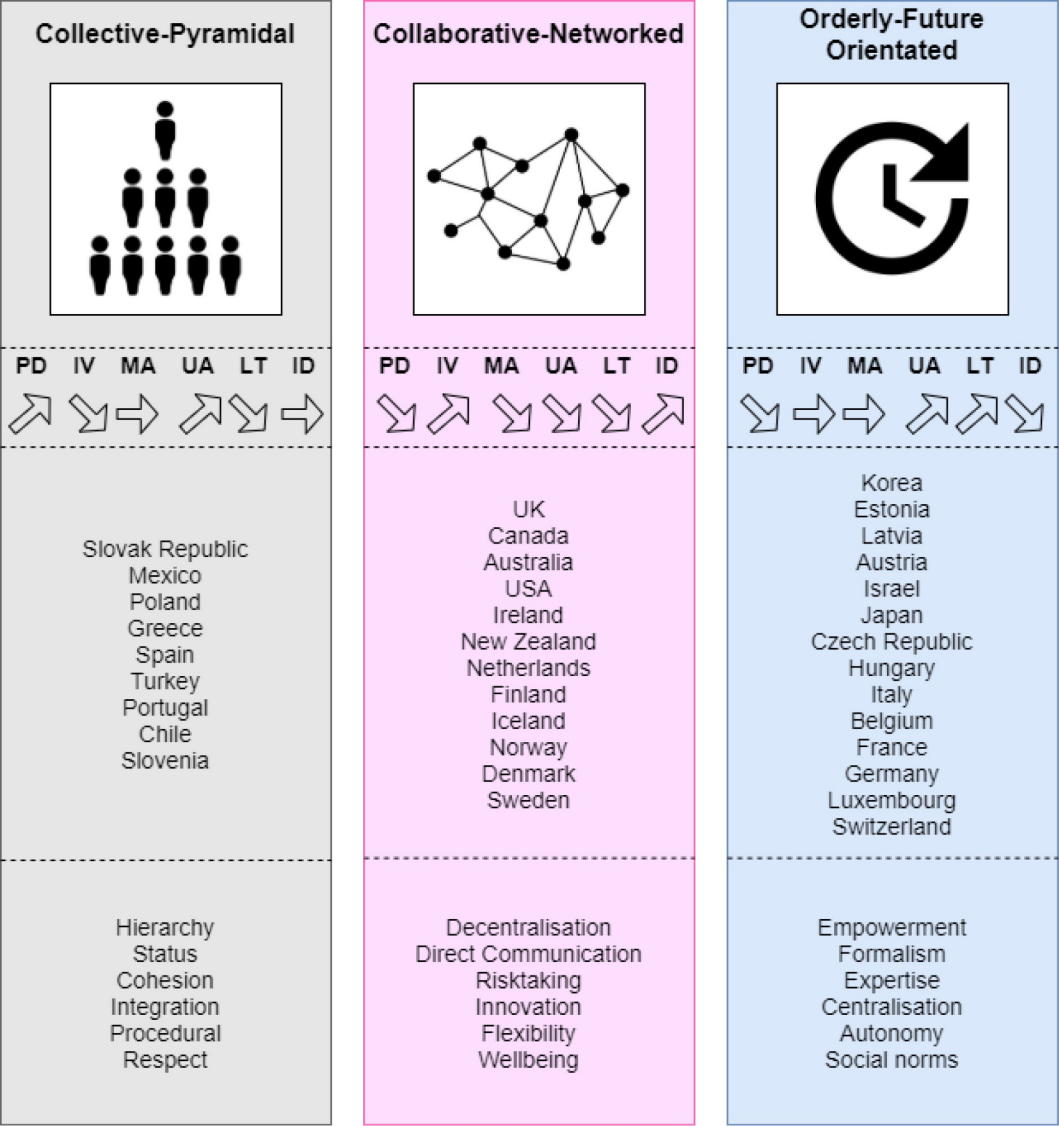
Illustrative description of the three national culture clusters identified (Figure adapted from Wursten [30]).

Descriptive statistics for the cultural dimensions by cluster groups are shown in Table 1. A one-way ANOVA found significant differences in all three clusters in the power distance index (F(2,32) = 21·2, p<0·001), individualism vs collectivism (F(2,32) = 21·0, p<0·001), uncertainty avoidance (F(2,32) = 35·3, p<0·001), long term vs short term orientation (F2,32) = 18·8, p<0·001) and indulgence vs restraint (F2,32) = 9·5, p = 0·001). However, there were no significant differences between the masculinity vs femininity dimension amongst the three clusters. We labelled and defined the black, pink and blue clusters as shown in Fig 3:

**Cluster 1: “Collective-Pyramidal”**: High power distance, collectivism, high uncertainty avoidance, short-term orientation.

**Cluster 2: “Collaborative-Networked”**: Low power distance, low uncertainty avoidance, individualism, indulgence.

**Cluster 3: “Orderly-Future Orientated”**: Low power distance, high uncertainty avoidance, long-term orientation, restraint.

**Table 1:** Descriptive statistics of cultural dimensions for the three clusters.

Cultural Dimension	Cluster	Mean	SD	95% CI
Power Distance	Collective-Pyramidal	69·9	13·3	59·7–80·1
	Collaborative-Networked	31·7	6·7	27·5–36·0
	Orderly-Future Orientated	44·1	17·2	34·1–54·0
Individualism vs Collectivism	Collective-Pyramidal	38·0	13·2	27·9–48·1
	Collaborative-Networked	76·3	10·3	69·8–82·9
	Orderly-Future Orientated	56·9	23·2	43·5–70·3
Masculinity vs Femininity	Collective-Pyramidal	50·6	25·0	31·3–69·8
	Collaborative-Networked	37·2	25·9	20·7–53·6
	Orderly-Future Orientated	56·9	23·2	43·5–70·3
Uncertainty Avoidance	Collective-Pyramidal	85·6	14·4	74·5–96·6
	Collaborative-Networked	44·0	10·9	37·1–50·9
	Orderly-Future Orientated	75·4	11·6	38·6–82·1
Long-term orientation	Collective-Pyramidal	42·9	15·8	30·8–55·0
	Collaborative-Networked	37·3	13·5	28·7–45·8
	Orderly-Future Orientated	70·9	15·5	61·9–79·8
Indulgence vs Restraint	Collective-Pyramidal	49·6	21·8	32·8–66·3
	Collaborative-Networked	67·6	6·5	63·5–71·7
	Orderly-Future Orientated	40·9	16·8	45·6–58·9

**Fig 4:**
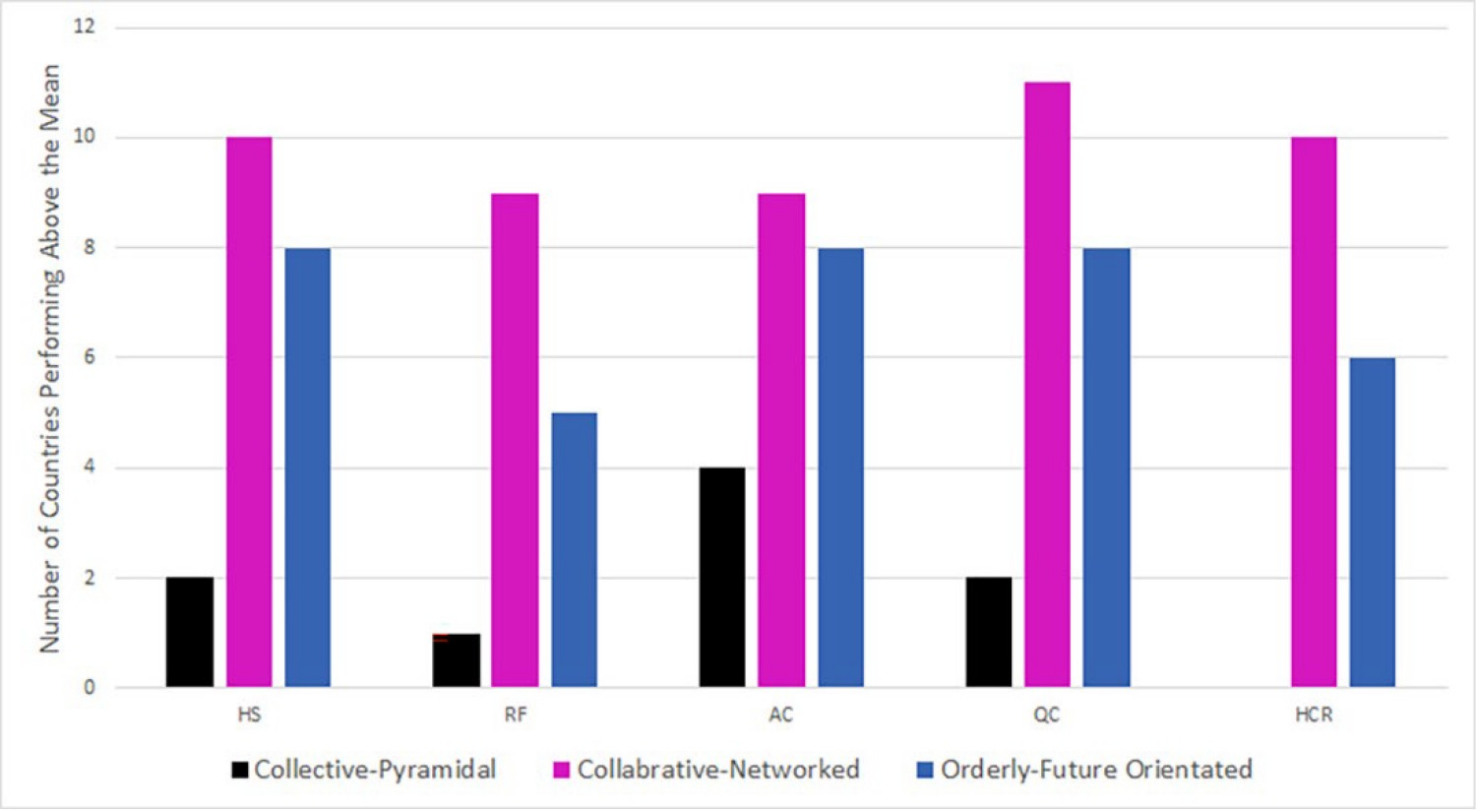
Number of countries within each cluster with performance above the mean on OECD healthcare system performance indicators.

Next, we present data on the relationship between the cultural dimensional cluster groups and the five OECD healthcare system performance indicators, summarised in Fig 4. Fisher’s exact test examined differences within and between the three cluster groups. Standardized scores of zero or greater indicated healthcare systems performance at or above the mean. Significant differences were found between cluster groups for health status, (Collective-Pyramidal = 22·2%, Collaborative-Networked = 83·3%, and Orderly-Future Orientated = 57·1% (p = 0·02)), risk factors (lower risk), (Collective-Pyramidal = 11·1%, Collaborative-Networked = 75·0%, Orderly-Future Orientated = 35·7% (p = 0·01)), quality of care (Collective-Pyramidal = 22·2%, Collaborative-Networked = 91·7% and Orderly-Future Orientated = 57·1% (p = 0·006)), and healthcare resources (Collective-Pyramidal = 0·0%, Collaborative-Networked = 83·3% and Orderly-Future Orientated = 42·9% (p<0·001)). There were no significant differences between cluster groups in the proportion performing above the mean for access to care (Collective-Pyramidal = 44·4%, Collaborative-Networked = 66·7% and Orderly-Future Orientated = 64·3% (p = 0·62)). The Collective-Pyramidal cluster had fewer countries that were performing at or above the mean on all five health dimensions, while Collaborative-Networked cluster had the highest proportion of countries in this category, with the Orderly-Future Orientated cluster in between the two (see Fig 4).

The relationship between the cultural dimension cluster groups and SDG3 meeting health goals was also examined. A one-way ANOVA found significant differences between the three clusters and their SDG3 percentage scores (F(2,32) = 9·38, p = 0·001). Table 2 shows the descriptive statistics for the relationships between the three clusters and the SDG3 percentage scores, and the number of countries from each cluster within each SDG3 dashboard colour grading (green, orange, red).

**Table 2:** Descriptive statistics between the three clusters and the SDG3 percentage scores, and SDG3 dashboard colour grading.

Cluster	SDG3% Score	SDG3 Dashboard “green”	SDG3 Dashboard “yellow”	SDG3 Dashboard “orange”	SDG3 Dashboard “red”
	Mean (SD)	No. (%)	No. (%)	No. (%)	No. (%)
Collective-Pyramidal	88·21 (3·64)	0 (0·0)	3 (33·3)	6 (66·7)	0 (0·0)
Collaborative-Networked	94·90 (1·88)	10 (83·3)	2 (16·7)	0 (0·0)	0 (0·0)
Orderly-Future Orientated	91·78 (4·38)	1 (7·1)	8 (57·1)	4 (28·6)	1 (7·1)

**Fig 5:**
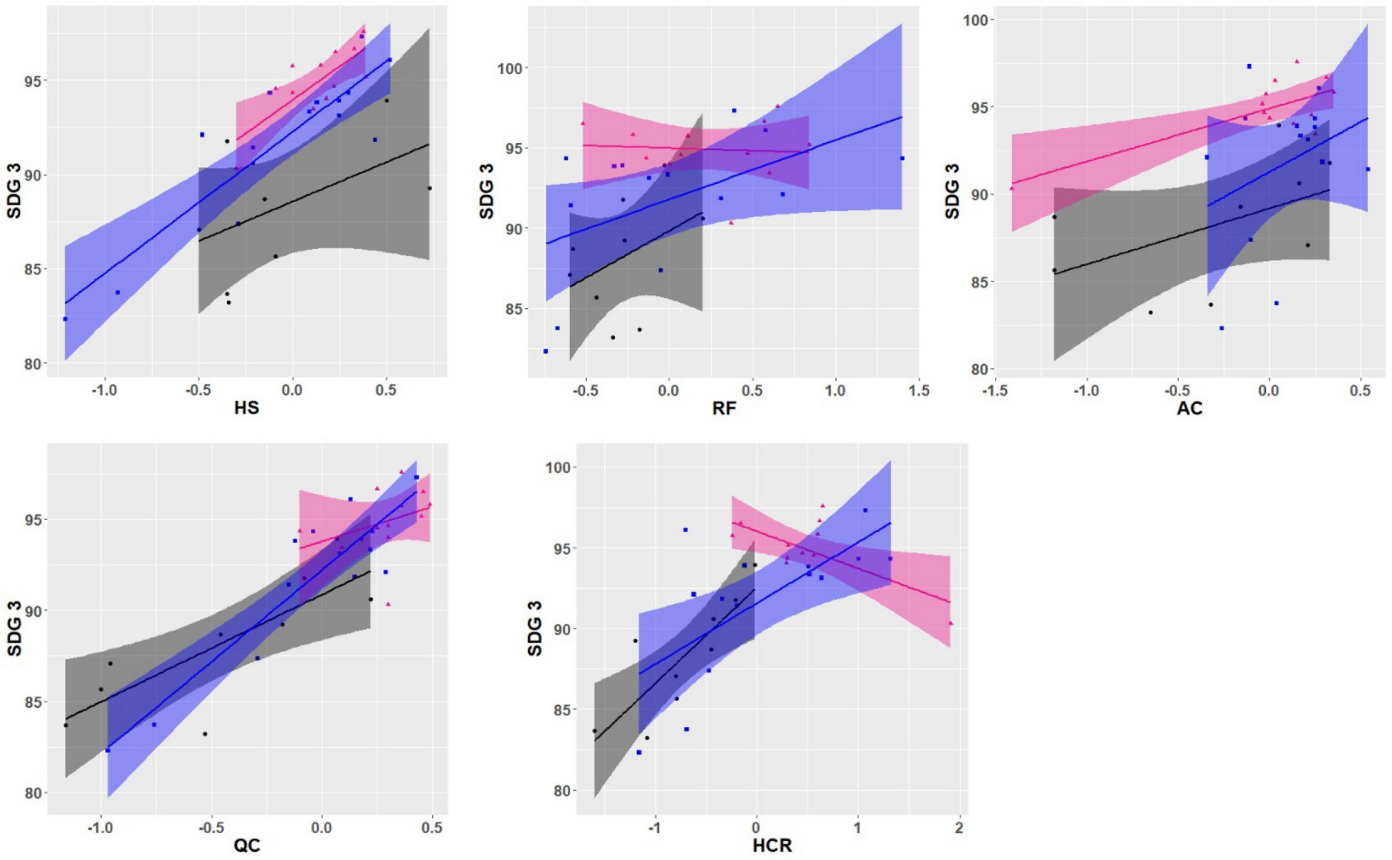
(a-e) Standardised scores for OECD performance indicators (a = HS, b = RF, c = AC, d = QC and e = HCR) with meeting health goals-SDG3 (% scores), between the three clusters (Collective-Pyramidal = black, Collaborative-Networked = pink, Orderly-Future Orientated = blue).

Fig 5A–5E show the relationships between OECD performance indicators (HS, RF, AC, QC and HCR) with meeting health goals, based on SDG3 percentage scores, for the three clusters. For the relationship between SDG3 percentages against standardised HS performance, positive linear relationships were observed in all three clusters. This was also observed for AC and QC performance indicators. For the relationship between SDG3 percentages against standardised RF performance, positive linear relationships were observed in the Collective-Pyramidal and Orderly-Future Orientated clusters; no linear relationship was observed in the Collaborative-Networked cluster. For the relationship between SDG3 percentages against standardised HCR performance, positive linear relationships were observed between the Collective-Pyramidal and Orderly-Future Orientated clusters, with a negative linear relationship holding for the Collaborative-Networked cluster.

## Discussion

### Overarching results: Cluster comparisons

We examined Hofstede’s national cultural dimensions and their relationships with national health performance indicators in 35 OECD countries, endeavouring to go inside the black box of performance and culture. Performance was measured via five OECD performance indicator groups and relative ranking of those countries against SDG3 performance outcomes. Country clusters emerged and were significantly different from each other on five of the six dimensions postulated by Hofstede. The relative performance of the country clusters was the same when measured by the OECD performance data and the SDG3 data: the Collaborative-Networked cluster performed best, followed by the Orderly-Future Orientated cluster, with the Collective-Pyramidal cluster running third.

Underscoring this finding, 10 of the twelve countries in the Collaborative-Networked cluster (83·3%) were classified as green, and thus rated as achieving SDG3. Only one country out of 14 (7·1%) from the Orderly-Future Orientated cluster was green, with eight of 14 (57·1%) in the yellow group, four of 14 (28·6%) in orange and one (7·1%) in red, indicating significant or major challenges remaining. No countries from the Collective-Pyramidal cluster were meeting SDG3, with three of nine (33·3%) and six of nine (66·7%) in yellow and orange respectively. These results provide evidence indicating a clear association between systems performance and national culture.

In terms of the comparative characteristics of the clusters, the Collaborative-Networked cluster, meeting SDGs, and the Collective-Pyramidal cluster, not meeting SDGs, were at either end of a continuum, with the Orderly-Future Orientated cluster’s results placing it more as a hybrid between the other two, with results that were more equivocal. In essence, the countries from the Orderly-Future Orientated cluster shared cultural characteristics that are a mix of both the Collective-Pyramidal and Collaborative-Networked cluster. For instance, the Collective-Pyramidal cluster can be described as having a high power distance index, more pronounced collectivism, and high uncertainty avoidance, whereas the Collaborative-Networked cluster in clear contrast has a low power distance index, stronger individualism and a low uncertainty avoidance. The Orderly-Future Orientated cluster’s cultural admixture of the other two exhibited low power distance index, mid-range but inclining towards individualism, and a high uncertainty index.

### The results in context: An antibiotic stewardship example

We wondered if there was further evidence to support or reject these findings. Amongst the many challenges of health systems, antibiotic stewardship is particularly problematic, with considerable efforts being expended across the OECD to ensure its judicious use [31]. Antibiotic use has been found to vary widely between countries. Antibiotic usage is a frequently used performance indicator by health policymakers as a measure of appropriate or inappropriate antibiotic prescribing. We found a relationship between national cultural clusters and performance on appropriate antibiotic prescribing. The cluster with low power distance index and low uncertainty avoidance, the Collective-Pyramidal cluster, had the lowest number of countries with performance above the mean in QC performance and lowest appropriate antibiotic prescribing with only 28.6% of countries performing above the mean. In comparison, 63·6% and 50% of the Collaborative-Networked and Orderly-Future Oriented clusters performed above the mean for appropriate antibiotic prescribing. Similarly, in other studies, the cultural dimensions on ‘power distance index’ and ‘uncertainty avoidance have been found to be positively correlated with antibiotic usage [21].

### Limitations

Our study, despite using multiple performance measures from differing sources and a well-established cultural model, and offering converging evidence for the phenomena found, has limitations. It is restricted to OECD countries, and may not generalise to other countries. Methodologically, while the data sources are robust and were gathered by trusted sources–the OECD and SDG3 –we were not party to the mechanisms and data cleansing activities that were executed to ensure their accuracy, efficacy and fidelity. It does not take into account institutional differences between countries; institutional arrangements are a factor; mediating systems-level performance, and remain under-investigated. While we clustered by culture and measured by the health indicators, future work might cluster using health indicators through methods such as Data Envelopment Analysis, where efficiency of the health system can be measured. Additionally, as is the case with studies of this kind, the data show associations not causation, and therefore cautious interpretation is recommended.

## Conclusion

Our study of the relationship between national cultures and health systems performance throws new light on how the two are associated. Countries within the Collaborative-Networked Cluster, characterised by low power distance, low uncertainty avoidance, individualism and indulgence, performed better than those in the other clusters. It is the case that these outcomes are mediated by institutional behaviour. Hofstede’s culture theory is an important way of construing performance and how it varies across countries. All-in-all, if ingrained national culture–practices, behaviours beliefs and mental models–are important variables in determining the performance of systems, this calls into question superficial change strategies, naive borrowing of policies and practices from one country to another, and linear or mechanistic views of systems improvement.
